# Comparison of Extended Total Extraperitoneal (E-TEP) Repair and Trans-Abdominal Pre-Peritoneal (TAPP) Mesh Repair in Inguinal Hernia Repair

**DOI:** 10.7759/cureus.39420

**Published:** 2023-05-24

**Authors:** Kancham Nethaji, Rinku Kumari, Pradeep Jaiswal, Pawan K Jha, Rajeev Ranjan, Ankur Akela

**Affiliations:** 1 General Surgery, Indira Gandhi Institute of Medical Sciences, Patna, IND; 2 Urology, Indira Gandhi Institute of Medical Sciences, Patna, IND

**Keywords:** extended total extraperitoneal repair, laparoscopic hernioplasty, trans-abdominal pre-peritoneal mesh repair, direct inguinal hernia, inguinal hernia repair

## Abstract

Background and aim: To find the superiority of extended total extraperitoneal (E-TEP) repair and trans-abdominal pre-peritoneal (TAPP) mesh repair in inguinal hernia repair.

Material and methods: A total of 30 patients with a unilateral or bilateral inguinal hernia (IH), and recurrent IH, following open repair were studied. Out of 30 patients, laparoscopic TAPP or E-TEP mesh repair was performed in an equal number of inguinal hernia patients. The patient's demographic parameters, duration of surgery, postoperative hospital stay, and complications were compared.

Results: In the E-TEP group, 33.33% of patients had left inguinal hernia (LIH), 60% of patients were diagnosed with right inguinal hernia (RIH) and 6.67% of patients had right inguinal and right direct hernia (RDH). In the TAPP group, 33.33% of patients had LIH and 53.33% of patients were suffering from RIH. Moreover, 6.67% of patients were diagnosed with a left inguinal direct hernia, and a similar proportion of patients had a right inguinal direct hernia. The mean duration of surgery was found to be significantly higher in the TAPP group (P<0.0000). The mean postoperative hospital stay was 2.07±0.59 and 2.80±1.32 days in E-TEP and TAPP groups, respectively (P=0.044).

Conclusion: In the present study, E-TEP mesh repair is a superior technique in the management of inguinal hernia as compared with TAPP repair.

## Introduction

“A hernia is a protrusion of viscus or a portion of a viscus through an abnormal opening in the walls of its containing cavities”. Inguinal hernia (IH) refers to the abdominal void protrusion via the inguinal canal. The “inguinal, femoral, and umbilical hernias”, which account for 75% of instances, are the most frequent types of external abdominal hernia [1]. According to estimates, the incidence of abdominal wall hernias is 1.7% for people of all ages and 4% for people over 45. Seventy-five percent of abdominal hernias are inguinal hernias (IHs), which have a lifetime risk of 27% in males and 3% in women [2,3].

The mainstay of diagnosis of IH is a clinical examination, and symptoms are typically suggestive. However, radio imaging may be required for the diagnosis of some hernias. Because of minor reappearance rates, mesh repair is the preferred method of treatment; nonetheless, herniorrhaphy is a popular, affordable option for many individuals. For pediatric patients, herniotomies and Mayo's repairs are the preferred techniques. Moreover, less postoperative morbidity and prompt arrival to work have made laparoscopic surgery more popular, although it is not very cost-efficient in rural settings [4].

Ger described performing the initial laparoscopic hernia restoration in the year 1982 by using stainless steel clips to approximate the internal ring [5]. Arregui et al. and Dion et al. proposed the “laparoscopic trans-abdominal pre-peritoneal (TAPP) repair”, a ground-breaking idea in hernia operation, in the early 90s [6,7]. Both the TAPP method and the extended total extraperitoneal (E-TEP) method can be used for laparoscopic groin hernia (GH) repair [7].

The inability to stratify patients for either E-TEP or TAPP repair has made the learning curve for laparoscopic repair of IHs even steeper. This is because there is a lack of reported results with respect to patient fulfillment, pain after the operation, length of hospital stays, problems, and hernia relapse. The purpose of the study is to equate the consequences of “laparoscopic hernioplasty” using the E-TEP method and TAPP method in order to ascertain whether the relative benefits achieved could be implemented on a large scale. The study also aims to identify criteria that may help stratify patients to different types of repairs in order to produce positive outcomes for each patient.

## Materials and methods

The present prospective observational study was conducted at the Department of Surgery, Indira Gandhi Institute of Medical Science, Sheikhpura, Patna, after institutional ethical committee approval (letter number: 1953/IEC/IGIMS/2020). A total of 30 patients of more than 18 years of age, diagnosed with unilateral or bilateral IH, or recurrent IH, and medically fit to undergo the procedure were recruited into the study. Patients with complicated IH and who require emergency exploration for complications of hernia like bowel obstruction, strangulation, gangrene, etc., and patients with failed laparoscopic repair of IH were excluded from the study.

A detailed study procedure was explained to the patients and relatives before enrolment. Written informed consent was obtained from the subjects prior to enrolment into the study. Out of 30 patients, 15 subjects were treated with laparoscopic TAPP mesh repair and 15 subjects were treated with E-TEP mesh repair of IH including follow-up of the patient done by randomized control study. TAPP and E-TEP procedures were performed by using polypropylene mesh. Laboratory investigations such as hemoglobin, total and differential leukocyte counts, platelet count, kidney function tests, liver function tests, prothrombin time, and international normalized ratio (INR) and radiology investigations such as x-ray and ultrasounds were performed. Patients were followed up at two weeks, eight weeks, and 10 weeks after surgery for any recurrences and complications.

Statistical analysis

Data were collected and evaluated by using SPSS V 1.2.5001 software (IBM Corp., Armonk, NY, USA). Continuous variables were expressed in terms of mean±SD, whereas categorical variables were presented as percentage and frequency. Normally distributed continuous variables were compared using the student T-test. Categorical variables were analyzed either by chi-square test or Fischer extract test. P<0.05 was considered statistically significant.

## Results

All patients were successfully recruited and completed the study. The mean age of patients in the E-TEP and TAPP groups was 42.67±7.25 years and 40.87±7.59 years, respectively (P=0.51). In the E-TEP group, the majority of patients were between 41 and 50 years of age (46.67%), while in the TAPP group, 46.67% belonged to the 31-40 year age group (Table 1). In both groups, RIH was the common diagnosis in the majority of the patients (Table 1).

**Table 1:** Distribution of subjects according to age and diagnosis Data presented as number (percentage) or mean±SD. E-TEP: extended total extraperitoneal repair; TAPP: trans-abdominal pre-peritoneal mesh repair; LIH: left inguinal hernia; RIH: right inguinal hernia; LDH: left direct hernia; RDH: right direct hernia.

Variables	Subcategories	E-TEP	TAPP	P value
Age (years)	≤30	1 (6.67)	2 (13.33)	0.91
	31-40	5 (33.33)	7 (46.67)	
	41-50	7 (46.67)	4 (26.67)	
	>50	2 (13.33)	2 (13.33)	
Mean age (years)		42.67±7.25	40.87±7.59	0.51
Diagnosis	LIH	5 (33.33)	5 (33.33)	0.23
	LIH + LDH	0 (0.00)	1 (6.67)	
	RIH	9 (60)	8 (53.33)	
	RIH + RDH	1 (6.67)	1 (6.67)	

Table 2 depicts the distribution of subjects according to postoperative pain score. Postoperative pain score between 1 and 2 was seen in eight (53.33%) and 10 (66.67%) patients of the E-TEP and TAPP groups, respectively (Table 2). In four (26.67%) and three (20%) patients of the E-TEP and TAPP groups, the pain score was 3-4. Postoperative pain score of 5-6 was observed in 20% and 13.33% of patients in E-TEP and TAPP groups, respectively (Table 2). There was no statistically significant difference in postoperative pain scores between the groups (P=0.75) (Table 2).

**Table 2:** The distribution of subjects according to postoperative pain score POP: postoperative pain; E-TEP: extended total extraperitoneal repair; TAPP: trans-abdominal pre-peritoneal mesh repair.

POP score	E-TEP		TAPP		P value
	Frequency (n)	Percentage (%)	Frequency (n)	Percentage (%)	
1-2	8	53.33	10	66.67	0.75
3-4	4	26.67	3	20.00	
5-6	3	20.00	2	13.33	
Total	15	100	15	100	

The mean duration of surgery in the E-TEP group was 78.33±8.16 minutes, ranging from 70 to 90 minutes (Table 3), whereas, in the TAPP group, the average duration of surgery was 99.73±9.81 minutes ranging from 85 to 120 minutes. A statistically significant difference in the mean duration of surgery was observed between the groups (P=0.00) (Table 3).

**Table 3:** Comparison of duration of the procedure E-TEP: extended total extraperitoneal repair; TAPP: trans-abdominal pre-peritoneal mesh repair; SD: standard deviation.

Duration of procedure (minutes)	E-TEP	TAPP	P value
Mean	78.33	99.73	0.00
SD	8.16	9.81	
Minimum	70	85	
Maximum	95	120	

In E-TEP and TAPP groups, the mean postoperative hospital stay was 2.07±0.59 days and 2.80±1.32 days, respectively (P=0.04) (Table 4). Table 5 depicts the incidence of complications in both groups. In the E-TEP group, shoulder pain and surgical emphysema were observed in one patient each. However, groin pain and port site infection were found in one and two patients of the TAPP group, respectively (Table 5).

**Table 4:** Mean postoperative hospital stay between the two groups *P<0.05. E-TEP: extended total extraperitoneal repair; TAPP: trans-abdominal pre-peritoneal mesh repair; SD: standard deviation.

Mean postoperative hospital stay	Mean±SD	P value
E-TEP (in days)	2.07±0.59	0.04*
TAPP (in days)	2.80±1.32	

**Table 5:** Distribution of subjects according to complications Data presented as number (percentage). E-TEP: extended total extraperitoneal repair; TAPP: trans-abdominal pre-peritoneal mesh repair.

Complications	E-TEP	TAPP
Groin pain	0 (0.00)	1 (6.67)
Port site infection	0 (0.00)	2 (13.33)
Shoulder pain	1 (6.67)	0 (0.00)
Surgical emphysema	1 (6.67)	0 (0.00)
Total	2 (13.33)	3 (20)

## Discussion

The present study aimed to find the superiority of E-TEP over TAPP techniques. The significant findings of the study were that the postoperative pain (POP) score was comparable between the groups. Duration of procedure was significantly more in TAPP group patients compared to E-TEP group patients. Postoperative complications were more in TAPP group patients compared to E-TEP group patients, but the difference was statistically insignificant. The duration of stay in the hospital was significantly high in TAPP group patients than in E-TAP patients.

POP is the most commonly observed complaint in IH surgery. Bilateral and IDHs are associated with increased POP [8,9]. In the present study, “POP” was evaluated by using the visual analog scale (VAS) score. Here, we observed no significant difference in pain scores between both groups (P=0.75). These findings are similar to the study by Sharma et al. and IEHS Guidelines 2011 [10,11]. Bansal et al. in their study reported less pain in TEP group patients compared to TAPP group patients [12]. Both groups reported having similar levels of chronic groin pain, despite the TAPP group reporting more acute pain. This could be attributed to the fact that the peritoneal incision heals with time and no longer affects the pain score.

In the current study, the duration of operation was significantly less in the E-TEP group compared to the TAPP group (78.33±8.16 minutes vs 99.73±9.81 minutes, P=0.00). These findings resemble the results of the study conducted by Bansal et al. and Rodha et al. [12-14]. The shorter surgical time required for E-TEP repair compared to TAPP repair may be because most of the cases in these studies involved unilateral hernias, and the initial space in E-TEP was filled with either a native glove finger balloon or a balloon dissector before the lateral space was only dissected. In unilateral hernias, this area affords E-TEP an operating time advantage over TAPP. Furthermore, few studies have reported an increased duration of surgery in E-TEP repair [10,15]. This might be because TEP repair for bilateral hernias, where the dissection must be transferred from one side to the other at the same working plane, requires less time to do than TAPP, which requires the creation of new flaps on both sides. The medial dissection (retro-pubic) across the midline is already finished from the first operated side; therefore, creating a new flap on the second operated side of TAPP also does not take very long.

In this study, one patient had groin pain and two patients had port site infections in the TAPP repair, whereas, in E-TEP repair, one patient had shoulder pain and one patient showed surgical emphysema. The incidence of complications is more in the TAPP repair as compared to the E-TEP repair. These findings are similar to the earlier reported studies by Singh et al. [16].

The duration of the patient's hospital stay increases the hospital's and the patient's financial burden. Because laparoscopic hernia surgery is a minimally invasive technique, it has a shorter recovery period than open hernia repair. In this study, the mean duration of hospital stay was remarkably extensive in the TAPP repair (2.80±1.32 days) as compared to the E-TEP repair (2.07±0.59 days) (P=0.04) which was similar to the results of Gass et al. and Bracale et al. [15,17]. Because of surgery for higher abnormalities and a higher percentage of complete hernia patients in the TAPP repair compared to the TEP repair, the TAPP group's longer hospital stays may be the result. However, prolonged hospital stays are a result of the higher incidence of complications.

In the present study minimum complications, postoperative hospital stays, and duration of operation were found to be significant factors in E-TEP repair which indicates that E-TEP mesh repair can be a superior technique in the management of IH when compared with TAPP repair. The limitations of the study involve the minimum sample size, and it was difficult to remark on the recurrences as the trial was short. Further, a multicenter study with a sufficient sample size including all the factors is a further recommendation of the study.

## Conclusions

The duration of surgery was significantly less in E-TEP repair than in TAPP repair. The incidence of complication was more in the TAPP group compared to the E-TEP group; however, the difference was statistically insignificant. The mean duration of hospital stay was more in TAPP group patients than in E-TEP group patients. E-TEP provides a better field of view and less incidence of intestinal injuries as compared to TAPP. However, bleeding is more common in the TEP technique. Therefore, it cannot be concluded that the E-TEP technique is superior to TAPP with the number of cases in this study and the complications described. So, further studies are warranted to confirm the present study findings.

## References

[1] Shakil A, Aparicio K, Barta E, Munez K (2020). Inguinal hernias: diagnosis and management. Am Fam Physician.

[2] Primatesta P, Goldacre MJ (1996). Inguinal hernia repair: incidence of elective and emergency surgery, readmission and mortality. Int J Epidemiol.

[3] Kingsnorth A, LeBlanc K (2003). Hernias: inguinal and incisional. Lancet.

[4] Pandya B, Huda T, Gupta D, Mehra B, Narang R (2021). Abdominal wall hernias: an epidemiological profile and surgical experience from a Rural Medical College in Central India. Surg J (N Y).

[5] Ger R (1982). The management of certain abdominal herniae by intra-abdominal closure of the neck of the sac. Preliminary communication. Ann R Coll Surg Engl.

[6] Arregui ME, Davis CJ, Yucel O, Nagan RF (1992). Laparoscopic mesh repair of inguinal hernia using a preperitoneal approach: a preliminary report. Surg Laparosc Endosc.

[7] Dion YM, Katkhouda N, Rouleau C, Aucoin A (1993). Laparoscopy-assisted aortobifemoral bypass. Surg Laparosc Endosc.

[8] McKernan JB, Laws HL (1993). Laparoscopic repair of inguinal hernias using a totally extraperitoneal prosthetic approach. Surg Endosc.

[9] Lal P, Philips P, Saxena KN, Kajla RK, Chander J, Ramteke VK (2007). Laparoscopic total extraperitoneal (TEP) inguinal hernia repair under epidural anesthesia: a detailed evaluation. Surg Endosc.

[10] Sharma D, Yadav K, Hazrah P, Borgharia S, Lal R, Thomas S (2015). Prospective randomized trial comparing laparoscopic transabdominal preperitoneal (TAPP) and laparoscopic totally extra peritoneal (TEP) approach for bilateral inguinal hernias. Int J Surg.

[11] Bittner R, Arregui ME, Bisgaard T (2011). Guidelines for laparoscopic (TAPP) and endoscopic (TEP) treatment of inguinal hernia [International Endohernia Society (IEHS)]. Surg Endosc.

[12] Bansal VK, Misra MC, Babu D (2013). A prospective, randomized comparison of long-term outcomes: chronic groin pain and quality of life following totally extraperitoneal (TEP) and transabdominal preperitoneal (TAPP) laparoscopic inguinal hernia repair. Surg Endosc.

[13] Rodha MS, Meena SP, Premi K, Sharma N, Puranik A, Chaudhary R (2022). Pain after transabdominal preperitoneal (TAPP) or totally extraperitoneal (TEP) technique for unilateral inguinal hernia: a randomized controlled trial. Cureus.

[14] Bansal VK, Krishna A, Manek P (2017). A prospective randomized comparison of testicular functions, sexual functions and quality of life following laparoscopic totally extra-peritoneal (TEP) and trans-abdominal pre-peritoneal (TAPP) inguinal hernia repairs. Surg Endosc.

[15] Gass M, Banz VM, Rosella L, Adamina M, Candinas D, Güller U (2012). TAPP or TEP? Population-based analysis of prospective data on 4,552 patients undergoing endoscopic inguinal hernia repair. World J Surg.

[16] Singh S, Kala S, Jauhari RK, Mishra Y, Yadav A (2022). A prospective randomized study of eTEP and TEP repair for inguinal hernia in terms of ease of operability, complication and recurrences. Asian J Med Sci.

[17] Bracale U, Melillo P, Pignata G, Di Salvo E, Rovani M, Merola G, Pecchia L (2012). Which is the best laparoscopic approach for inguinal hernia repair: TEP or TAPP? A systematic review of the literature with a network meta-analysis. Surg Endosc.

